# A Topical Formulation of Melatoninergic Compounds Exerts Strong Hypotensive and Neuroprotective Effects in a Rat Model of Hypertensive Glaucoma

**DOI:** 10.3390/ijms21239267

**Published:** 2020-12-04

**Authors:** Massimo Dal Monte, Maurizio Cammalleri, Rosario Amato, Salvatore Pezzino, Roberta Corsaro, Paola Bagnoli, Dario Rusciano

**Affiliations:** 1 Department of Biology, University of Pisa, via San Zeno, 31, 56127 Pisa, Italy; maurizio.cammalleri@unipi.it (M.C.); rosario.amato@biologia.unipi.it (R.A.); paola.bagnoli@unipi.it (P.B.); 2 Sooft Research Center c/o, University of Catania, Via Santa Sofia, 89, 95123 Catania, Italy; salvatore.pezzino@sooft.it (S.P.); roberta.corsaro@sooft.it (R.C.); dario.rusciano@sooft.it (D.R.)

**Keywords:** intraocular pressure, timolol, brimonidine, melatonin/agomelatine, electroretinography, retinal ganglion cells, gliosis/inflammation, apoptotic cascade

## Abstract

Melatonin is of great importance for regulating several eye processes, including pressure homeostasis. Melatonin in combination with agomelatine has been recently reported to reduce intraocular pressure (IOP) with higher efficacy than each compound alone. Here, we used the methylcellulose (MCE) rat model of hypertensive glaucoma, an optic neuropathy characterized by the apoptotic death of retinal ganglion cells (RGCs), to evaluate the hypotensive and neuroprotective efficacy of an eye drop nanomicellar formulation containing melatonin/agomelatine. Eye tissue distribution of melatonin/agomelatine in healthy rats was evaluated by HPLC/MS/MS. In the MCE model, we assessed by tonometry the hypotensive efficacy of melatonin/agomelatine. Neuroprotection was revealed by electroretinography; by levels of inflammatory and apoptotic markers; and by RGC density. The effects of melatonin/agomelatine were compared with those of timolol (a beta blocker with prevalent hypotensive activity) or brimonidine (an alpha 2 adrenergic agonist with potential neuroprotective efficacy), two drugs commonly used to treat glaucoma. Both melatonin and agomelatine penetrate the posterior segment of the eye. In the MCE model, IOP elevation was drastically reduced by melatonin/agomelatine with higher efficacy than that of timolol or brimonidine. Concomitantly, gliosis-related inflammation and the Bax-associated apoptosis were partially prevented, thus leading to RGC survival and recovered retinal dysfunction. We suggest that topical melatoninergic compounds might be beneficial for ocular health.

## 1. Introduction

Knowledge in the biology of melatonin, an indoleamine with potent multifunctional biological and pharmacological effects, is increasing day by day although much work remains to be done in order to explore its potential therapeutic use. Melatonin is the main hormone secreted by the pineal gland following a circadian rhythm, with low levels during the day and elevated levels at night [1]. Extrapineal sources of melatonin were reported in different tissues and organs including the retina in which melatonin is synthetized and released rhythmically by retinal photoreceptors and ciliary epithelium [2]. In the retina, melatonin plays as an oxyradical scavenger and exerts a strong antioxidative and anti-inflammatory action [3]. This natural indoleamine is of great importance for regulating several eye processes, among which pressure homeostasis is included [4].

Glaucoma, the most prevalent eye disease, is a heterogeneous group of eye disorders with major oxidative/inflammatory components, which is associated to age and, often, to increased intraocular pressure (IOP). Glaucoma is an optic neuropathy characterized by the chronic and progressive apoptotic death of retinal ganglion cells (RGCs), which are located in the inner retina. Their axons merge to form the optic nerve and progressively degenerate as a consequence of increased IOP at the optic nerve head [5]. Progressive RGC loss in glaucoma is a leading cause of irreversible blindness, second only to macular degeneration. Glaucoma is a major public health problem, as it is a leading cause of blindness worldwide; among different types of glaucoma, primary open angle glaucoma accounts for about 74% of all glaucoma cases [6]. Although the pathogenesis of the disease is unclear, IOP elevation, caused by an imbalance in the production and drainage of aqueous humor in the anterior chamber of the eye, is considered one of the main risk factors in glaucoma development and progression [5]. Indeed, IOP is the only modifiable risk factor and, as such, medications are available to control it; however, novel medications are sought to counteract other risk factors that, in addition to IOP elevation, participate to RGC loss. This is confirmed by the fact that in normal-tension glaucoma, the damage to the optic nerve occurs even though eye pressure is not elevated and that in hypertensive glaucoma the death of RGCs may continue despite IOP reduction [7,8].

In hypertensive glaucomatous retina, IOP elevation rapidly activates the combined response of reactive gliosis and related inflammation as demonstrated by an early induction of pro-inflammatory mediators and increased glial fibrillary acidic protein (GFAP) levels [9]. Gliosis-related inflammation in the retina activates Bax-caspase-3-dependent apoptotic pathways that result in RGC degeneration by apoptosis [10]. Within this framework, topical treatments that can be well tolerated by patients and that, possibly, may counteract IOP elevation and protect RGCs from death would be of great help for glaucoma patient compliance. Currently, topical ocular hypotensive drugs as well as surgical approaches are the cutting-edge for IOP reduction [11].

Among IOP-lowering drugs used clinically for the treatment of glaucoma and ocular hypertension, the beta blocker timolol significantly reduces IOP increase, but has a marginally neuroprotective capability that follows IOP reduction [12]. An additional IOP-lowering drug is the selective alpha 2 adrenergic agonist brimonidine commonly used as eyedrop for short- and long-term lowering of IOP although its use is limited by a relatively high rate of ocular allergy, hyperemia and discomfort. The therapeutic use of brimonidine has been popularized due to its effects on aqueous suppression and uveoscleral outflow, as well as the suggestion of neuroprotection. The neuroprotective efficacy of brimonidine has been demonstrated by preclinical and clinical evidence showing that brimonidine may exert a neuroprotective role on RGCs independently from its hypotensive efficacy [13,14]. Presently, the potential of additional topical treatments that can be well tolerated by patients and that, possibly, may counteract both IOP elevation and RGC degeneration has been documented in preclinical models, although their current therapeutic use is limited by the lack of definitive results in clinical trials [15,16].

Melatonin and its analogues have been reported to decrease IOP in both normotensive and hypertensive eyes [17,18,19,20]. Recently, we have shown that a combination of melatonin and its analogue methoxycarbonylamino-*N*-acetyltryptamine, or agomelatine (commonly used as a systemic anti-depressant), exerts hypotensive activity in normotensive eyes of the rat and in a rat model of IOP elevation. We reported that nanomicellar formulations of melatonin and agomelatine, either alone or in combination, had lowering effects that did not depend on their concentration or their combination, which, however, resulted in an increased duration of the hypotensive efficacy [17]. In addition, in a rat model of hypertensive glaucoma in which IOP elevation is induced by the injection of methylcellulose (MCE) in the anterior chamber of the eye, we showed that topical administration of a nanomicellar formulation of melatonin/agomelatine drastically reduces IOP elevation with an increased duration of the hypotensive effect by the combination of the two melatoninergic agents in respect to each agent alone [17]. Although melatonin levels have been found to increase in the aqueous humor of glaucoma patients, possibly reflecting a mechanism of protection triggered by IOP increase [21], clinical evidence of the hypotensive efficacy of melatoninergic agents is limited to the demonstration that oral administration of the melatonin analogue agomelatine reduces IOP in glaucoma patients [22]. Of note, besides exerting hypotensive activity, additional potential of melatonin as neuroprotectant has been demonstrated in rodent models of retinal diseases [23,24,25,26,27]. In addition, melatonin treatment has been shown to increase RGC survival in experimental models of RGC death suggesting it as a promising neuroprotective agent for the treatment of ocular neurodegenerative diseases [27,28]. Of note, melatonin subcutaneously implanted in a rat model of hypertensive glaucoma does not affect IOP but prevents IOP-induced RGC death and retinal dysfunction [29], suggesting that melatoninergic agents may exert neuroprotective effects independently of their hypotensive activity. On the other hand, in the glaucoma model of episcleral vein cauterization, melatonin fails to exert a neuroprotective effect on RGCs although its administration results in decreased IOP [30].

In the present study, we used the MCE model of glaucoma to investigate whether an eye drop nanomicellar formulation containing melatonin and agomelatine may protect RGCs from apoptotic death. In healthy rats, we first evaluated the distribution of the melatoninergic compounds in the whole eye, the vitreous body and the retina by HPLC/MS/MS. In the MCE model of glaucoma, IOP was monitored up to two weeks after MCE injection when electrophysiological evaluation of retinal function was performed in order to isolate RGC responses from the electroretinographic activity. To this aim, the photopic negative response (PhNR), a negative-going wave following the b-wave of the cone electroretinogram (ERG), and the pattern ERG (PERG) in response to contrast-reversing gratings, both indicative of RGC activity [31], were recorded to evaluate whether melatoninergic compounds may counteract retinal dysfunction consequent to RGC death induced by IOP elevation. Whether melatonin/agomelatine affects markers of gliosis-related inflammation was also investigated in retinal homogenates by Western blot analysis. In addition, Western blot for Bax, Bcl-2, and caspase-3 was used to explore the Bax-associated apoptotic pathways leading to RGC death. In this respect, we determined whether hypotensive efficacy of melatoninergic compounds is associated to RGC protection by quantitating RGC survival using immunohistochemistry for brain-specific homeobox/POU domain protein 3A (Brn3a), a transcription factor that is considered a specific and reliable marker of RGCs [32]. Hypotensive and neuroprotective effects of the melatoninergic agents were compared with those of two well characterized anti-glaucoma drugs, timolol or brimonidine, for a further dissection of the findings from melatonin and agomelatine, e.g., corroborating their apparent hypotensive and neuroprotective effects with those of timolol (with prevalent hypotensive activity) or brimonidine (with neuroprotective efficacy independent on its hypotensive effects). Prostaglandin analogues, a class of hypotensive drugs widely used in the treatment of hypertensive glaucoma, have not been considered in this study because they have not been thoroughly characterized in terms of neuroprotection activity in experimental model systems, and one study comparing the effects of systemic brimonidine with latanoprost eye drops showed an IOP independent neuroprotective activity of brimonidine, and negligible neuroprotection by topical latanoprost [33].

## 2. Results

### 2.1. Melatonin/Agomelatine in the Eye

The quantitative analysis by HPLC/MS/MS (Table 1) revealed that of the 120 µg given in 3 administrations of 40 μg each at a distance of 2 h as 10 µL eye drops, roughly 1% of melatonin (1.19 µg) and 1.3% of agomelatine (1.59 µg) were retained in the whole eye. When looking at the amount detectable in the vitreous body, we found an average concentration of 0.01 ng/mg of melatonin and 0.08 ng/mg of agomelatine. In the retina the concentration of melatonin was much lower, amounting to 0.004 ng/mg, while agomelatine was slightly higher, at 0.096 ng/mg. Such concentrations in the retina roughly correspond to 17 nM and 378 nM, respectively, for melatonin and agomelatine, all available for receptor binding.

### 2.2. Effects on IOP Elevation

**Figure 1:**
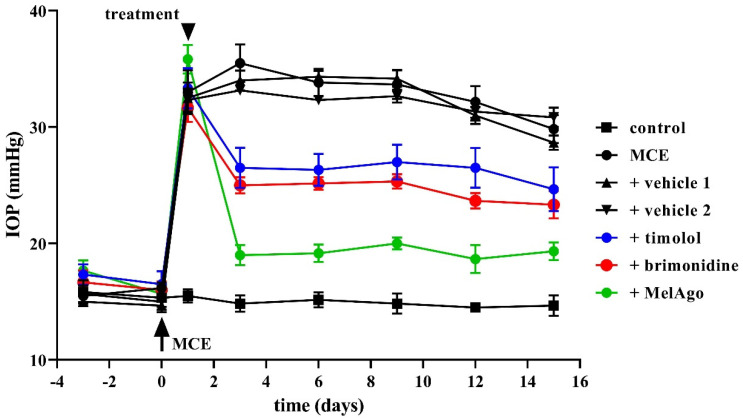
Effects of topical treatments on intraocular pressure (IOP) in a rat model of hypertensive glaucoma. Methylcellulose (MCE) injection in the anterior chamber at day 0 (arrow) significantly increased IOP (*p* < 0.05; two-way ANOVA followed by Bonferroni’s multiple comparison post-test). Treatments started at day 1 (arrowhead). Vehicles (saline: vehicle 1, and nanomicellar formulation: vehicle 2) did not affect IOP, which was instead significantly reduced by timolol, brimonidine, or melatonin/agomelatine (*p* < 0.05; two-way ANOVA followed by Bonferroni’s multiple comparison post-test). The hypotensive effect of melatonin/agomelatine was greater than that of timolol or brimonidine (*p* < 0.05; two-way ANOVA followed by Bonferroni’s multiple comparison post-test). Data are shown as mean ± SEM (*n* = 9 rats, 18 eyes for each experimental group). MelAgo, melatonin/agomelatine.

As shown in Figure 1, IOP increased 24 h after MCE injection in line with previous findings [17,34,35,36]. IOP elevation was maintained up to 14 days (from 16.2 ± 1.7 at day 0 to 33.0 ± 4.6 mmHg starting from day 1). Both vehicles (saline: vehicle 1, and nanomicellar formulation: vehicle 2) did not affect IOP increase. Two weeks after MCE injection, an IOP decrease of about 10% could be noticed likely due to the reduction over time of the hypertensive effect of MCE [36]. IOP elevation was significantly reduced by timolol, brimonidine, or melatonin/agomelatine. The IOP-lowering effect of melatoninergic compounds was greater than that of either timolol or brimonidine. After two weeks of administration, timolol or brimonidine reduced the MCE-induced increase in IOP by about 32% or 34% in respect to vehicle 1, while melatonin/agomelatine reduced the MCE-induced increase in IOP by about 60% in respect to vehicle 2, with an effect that was about twice than that of timolol or brimonidine.

### 2.3. Effects on Retinal Dysfunction: PhNR and PERG

**Figure 2:**
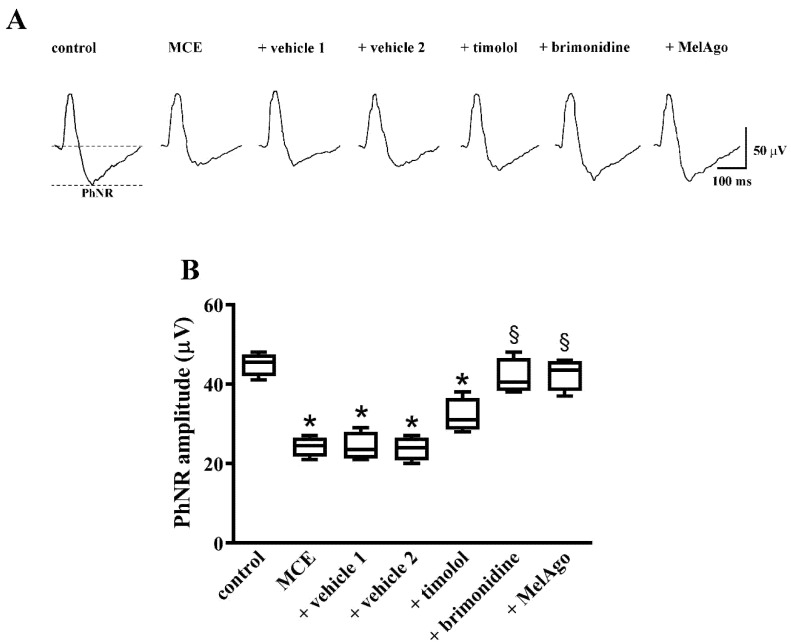
Retinal function as evaluated by photopic full-field electroretinogram (ERG). (**A**) Representative ERG traces showing photopic b-waves with photopic negative response (PhNR) in control rats and in rats that received intraocular MCE injection either untreated or treated with vehicles, timolol, brimonidine or melatonin/agomelatine. (**B**) Mean amplitudes of PhNR evaluated as changes from baseline. MCE reduced the PhNR amplitude. Neither vehicles nor timolol affected the PhNR amplitude that was almost recovered to control values by brimonidine or melatonin/agomelatine. Data are shown as box and whiskers plots (*n* = 6 animals for each experimental group). * *p* < 0.05 versus control; ^§^ *p* < 0.05 versus the respective vehicle (one-way ANOVA followed by Tukey post-hoc test). MelAgo, melatonin/agomelatine.

As shown in Figure 2, in MCE-injected rats either untreated or treated with vehicles, the photopic b-wave did not differ in amplitude from controls. In contrast, the amplitude of the PhNR, as measured from the pre-stimulus baseline to the trough of the response, was reduced by about 50%, a reduction that is in line with previous studies indicating a selective loss of inner retinal function in glaucoma models [37,38]. In MCE-injected rats treated with timolol, PhNR amplitude did not differ from that measured after vehicle 1. In contrast, treatments with brimonidine or melatonin/agomelatine increased PhNR amplitude with regards to their respective vehicles thus almost restoring the PhNR amplitude to control levels.

**Figure 3:**
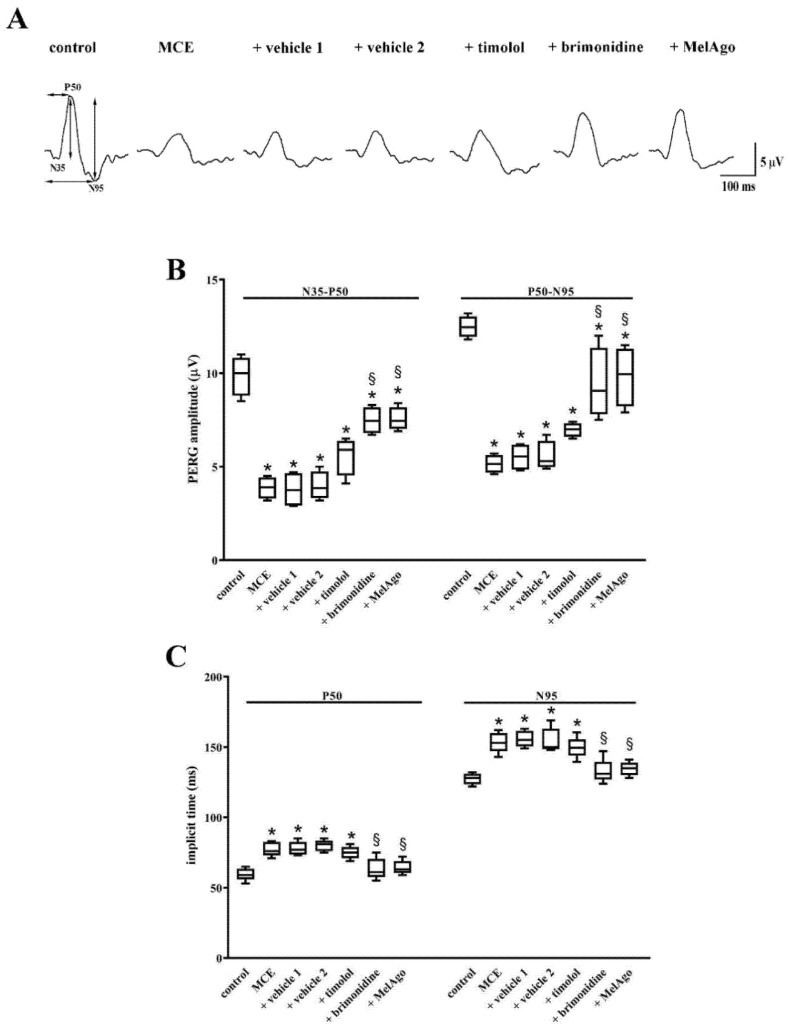
Retinal function as evaluated by pattern ERG (PERG). (**A**) Representative PERG traces showing the two negative peaks (N35 and N95) and the positive peak P50 in control rats and in rats that received intraocular MCE injection either untreated or treated with vehicles, timolol, brimonidine or melatonin/agomelatine. Vertical arrows indicate the amplitude of the N35-P50 and of the P50-N95 waves (from the through of the negative peak N35 to the top of the positive peak P50 and from the top of the positive peak P50 to the through of the negative peak N95, respectively). Horizontal arrows indicate the P50 and N95 implicit times (time from the onset of the stimulus to the P50 and N95 peaks, respectively). (**B**) Mean amplitudes of the N35–P50 and P50–N95 waves. MCE reduced the amplitude of both waves. Their amplitude was unaffected by vehicles or timolol, while it was partially restored by brimonidine or melatonin/agomelatine. (**C**) Mean implicit time of the P50 and N95 peaks. MCE increased the implicit times. Neither vehicles nor timolol affected the implicit times that were almost recovered by brimonidine or melatonin/agomelatine. Data are shown as box and whiskers plots (*n* = 6 animals for each experimental group). * *p* < 0.05 versus control; ^§^ *p* < 0.05 versus the respective vehicle (one-way ANOVA followed by Tukey post-hoc test). MelAgo, melatonin/agomelatine.

As shown in Figure 3, in MCE-injected rats either untreated or treated with vehicles, the amplitude of the N35-P50 and the P50-N95 waves of the PERG was reduced by about 60% and 55%, respectively. In rats treated with timolol, the amplitude of the N35-P50 and P50-N95 waves did not differ from that measured in rats treated with vehicle 1. In contrast, both brimonidine and melatonin/agomelatine partly prevented the reduction in the amplitudes of the N35-P50 and the P50-N95 waves. In particular, after brimonidine, the amplitudes of the N35-P50 and the P50-N95 waves were about 24% and 25% lower than in controls. After melatonin/agomelatine the amplitudes of the N35-P50 and the P50-N95 waves were about 23% and 21% lower than in controls. In rats treated with MCE either alone or in the presence of vehicles, the implicit time of the P50 and N95 components increased by about 28% and 21%, respectively. Timolol did not affect the MCE-induced increase in the implicit time that, in contrast, was prevented by both brimonidine and melatonin/agomelatine.

### 2.4. Effects on Gliosis-Related Inflammation

**Figure 4:**
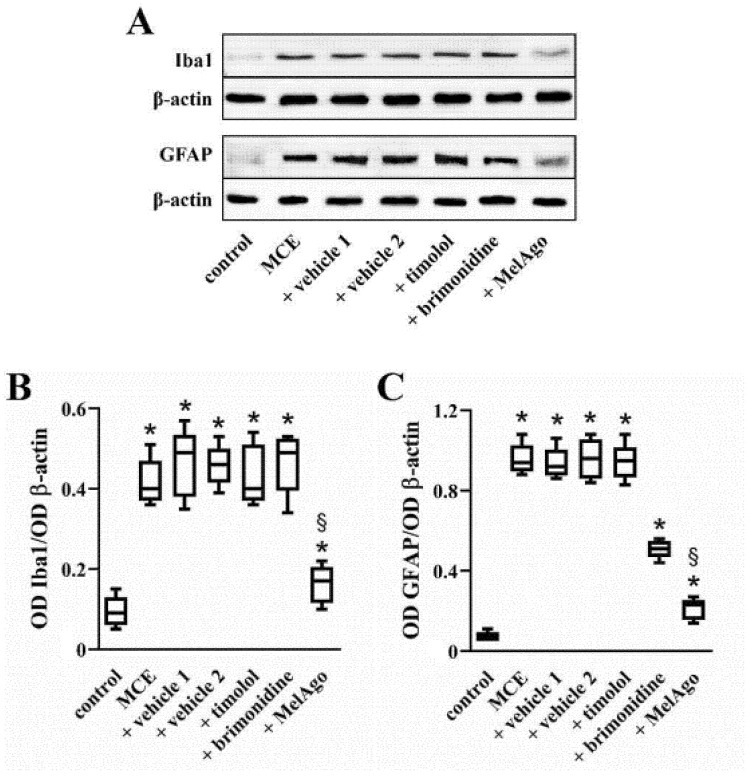
Glial activation. (**A**) Representative Western blots from retinal homogenates of control rats or rats that received MCE, either untreated or treated with vehicles, timolol, brimonidine, or melatonin/agomelatine. (**B**,**C**) Densitometric analysis of ionized calcium binding adaptor molecule 1 (Iba1; **B**) and glial fibrillary acidic protein (GFAP; **C**). MCE increased the levels of both Iba1 and GFAP. Iba1 and GFAP increase was unaffected by vehicles or timolol. Brimonidine reduced GFAP increase, but not Iba1 increase. Melatonin/agomelatine reduced both Iba1 and GFAP increase. Data are shown as box and whiskers plots (*n* = 6 samples, each containing two retinas, for each experimental group). * *p* < 0.05 versus control; ^§^ *p* < 0.05 versus the respective vehicle (one-way ANOVA followed by Tukey post-hoc test). MelAgo, melatonin/agomelatine.

As shown in Figure 4, IOP elevation following MCE injection induced a drastic increase in the levels of ionized calcium binding adaptor molecule 1 (Iba1, a microglia/macrophage-specific calcium-binding protein that participates in membrane ruffling and phagocytosis in activated microglia) and of GFAP (an intermediate filament protein that, in response to injury, is drastically up-regulated in Müller cells, the principal glial cells in the retina). In particular, Iba1 levels were increased by about 3.7-fold, while GFAP levels were increased by about 8.1-fold. Comparable levels were found in the presence of vehicles. Timolol did not affect the levels of Iba1 and GFAP, whereas brimonidine reduced the levels of GFAP by about 1.6-fold while not affecting Iba1 levels. Melatonin/agomelatine reduced the levels of both Iba1 and GFAP by about 2.3- and 3.2-fold, respectively.

**Figure 5:**
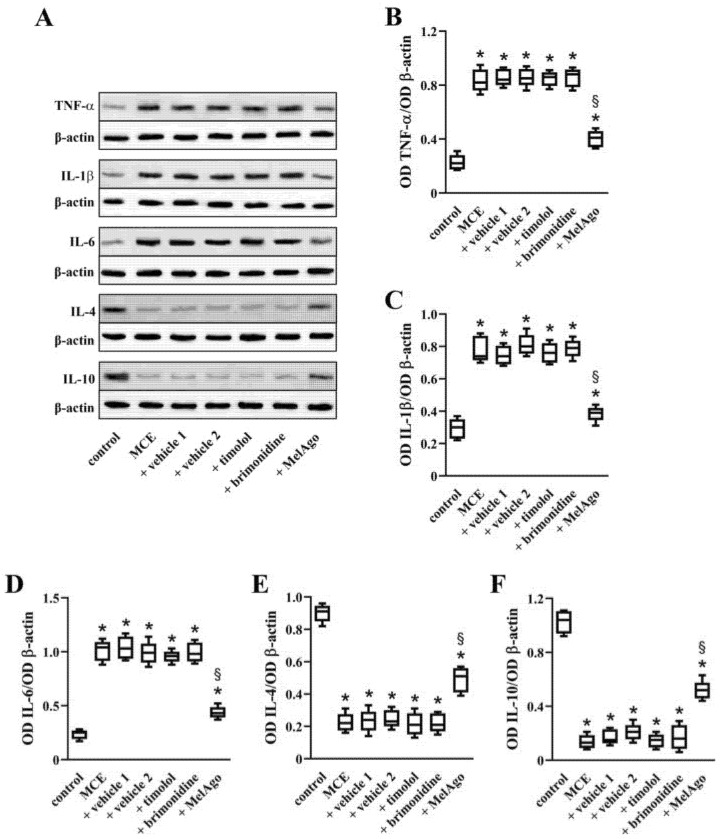
Levels of inflammatory markers. (**A**) Representative Western blots from retinal homogenates of control rats or rats that received MCE, either untreated or treated with vehicles, timolol, brimonidine or melatonin/agomelatine. (**B**–**F**) Densitometric analysis of the levels of tumor necrosis factor (TNF)-α (**B**), interleukin (IL)-1β (**C**), IL-6 (**D**), IL-4 (**E**), and IL-10 (**F**). After MCE, the levels of TNF-α, IL-1β and IL-6 were increased, while the levels of IL-4 and IL-10 were decreased. Vehicles, timolol or brimonidine did not affect the MCE-induced changes of the inflammatory markers, while melatonin/agomelatine almost restored their control levels. Data are shown as box and whiskers plots (*n* = 6 samples, each containing 2 retinas, for each experimental group). * *p* < 0.05 versus control; ^§^ *p* < 0.05 versus vehicle 2 (one-way ANOVA followed by Tukey post-hoc test). MelAgo, melatonin/agomelatine.

The activation of inflammatory processes by gliosis results in dysregulated production of cytokines that impacts on the apoptotic cascade leading to RGC loss [39]. As shown in Figure 5, MCE increased the levels of pro-inflammatory cytokines and decreased the levels of anti-inflammatory cytokines. In particular, the levels of the pro-inflammatory cytokines tumor necrosis factor (TNF)-α, interleukin (IL)-1β and IL-6 were upregulated (by about 3.7-, 2.7-, and 4.3-fold, respectively), while the levels of the anti-inflammatory cytokines IL-4 and IL-10 were downregulated (by about 4.0- and 7.5-fold, respectively). Comparable levels were determined in the presence of vehicles. Timolol and brimonidine did not affect the levels of both pro-inflammatory and anti-inflammatory cytokines. On the contrary, melatonin/agomelatine reduced the levels of TNF-α, IL-1β and IL-6 (by about 2.0-, 2.1-, and 2.3-fold, respectively), while increased the levels of IL-4 and IL-10 (by about 2.0- and 2.5-fold, respectively).

### 2.5. Effects on Bax-Caspase-3-Dependent Apoptotic Pathway

**Figure 6:**
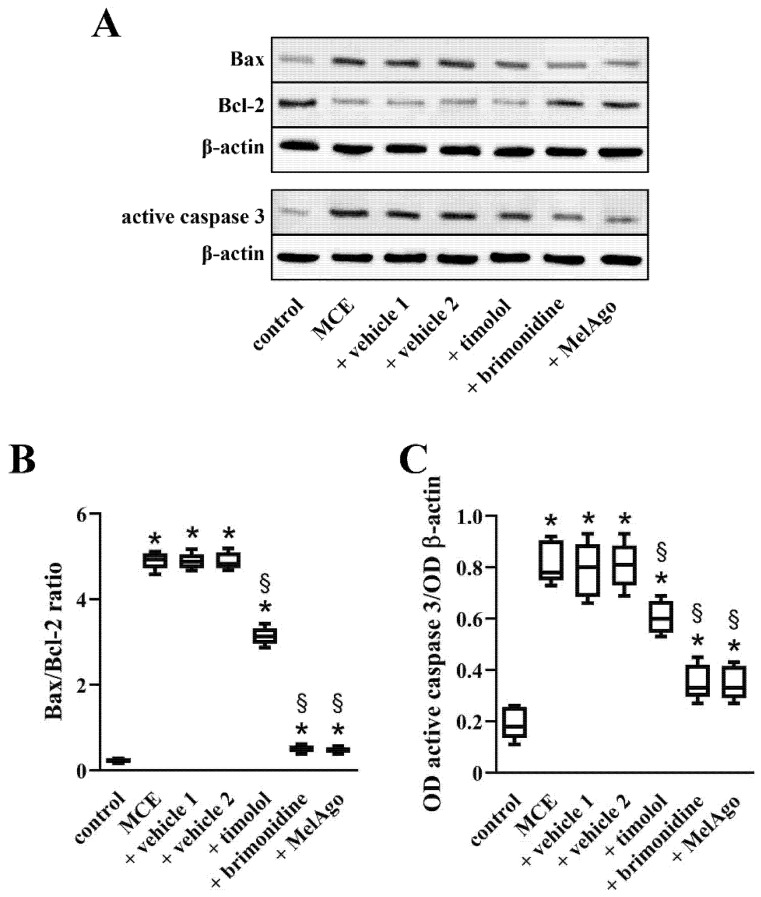
The Bax-caspase-3-dependent apoptotic pathway. (**A**) Representative Western blots from retinal homogenates of control rats or rats that received MCE, either untreated or treated with vehicles, timolol, brimonidine or melatonin/agomelatine. (**B**,**C**) Densitometric analysis of the levels of Bax/Bcl-2 (**B**) and active caspase 3 (**C**). MCE increased the Bax/Bcl-2 ratio and the level of active caspase 3. Vehicles did not affect the MCE-induced changes in apoptotic markers that were, in contrast, reduced by timolol, brimonidine, or melatonin/agomelatine, with a greater efficacy of brimonidine and melatonin/agomelatine. Data are shown as box and whiskers plots (*n* = 6 samples, each containing 2 retinas, for each experimental group). * *p* < 0.05 versus control; ^§^ *p* < 0.05 versus the respective vehicle (one-way ANOVA followed by Tukey post-hoc test). MelAgo, melatonin/agomelatine.

In glaucomatous eyes, gliosis-related inflammation activates common molecular signals that trigger the Bax-caspase-3-dependent apoptotic pathway [10]. As shown in Figure 6, MCE induced an increase of apoptotic markers, including the Bax/Bcl-2 ratio and the levels of the active form of caspase 3 by about 21.5- and 4.3-fold, respectively. Comparable levels were determined in the presence of vehicles. All the treatments were able to counteract apoptotic processes with a greater effectiveness of brimonidine and melatonin/agomelatine. In particular, the Bax/Bcl-2 ratio was reduced by about 1.6-fold by timolol, 9.7-fold by brimonidine and 10.3-fold by melatonin/agomelatine. The levels of the active form of caspase 3 were decreased by about 1.3-fold by timolol, 2.2-fold by brimonidine and 2.3-fold by melatonin/agomelatine.

### 2.6. Effects on RGCs Loss

**Figure 7:**
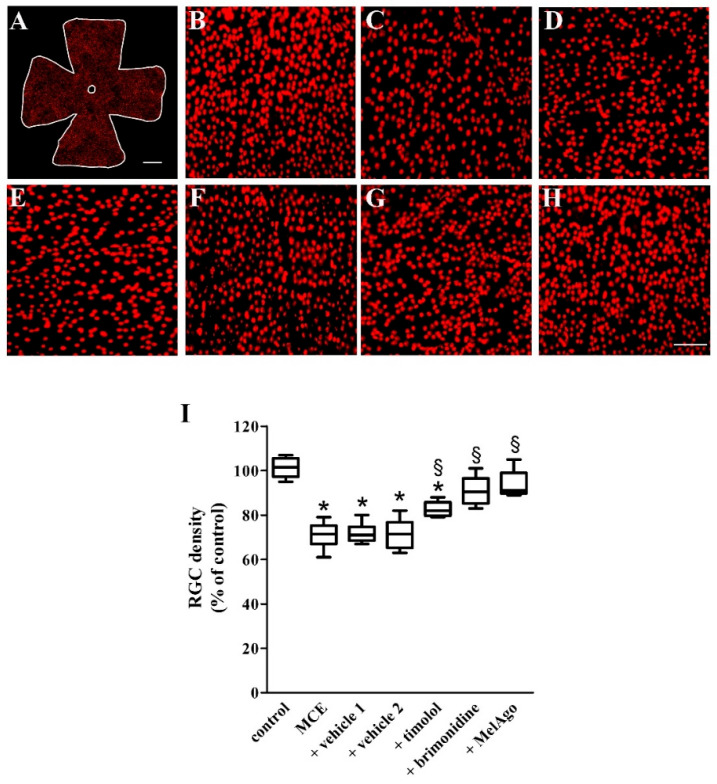
Retinal ganglion cell (RGC) density. (**A**) Representative image of a control flat mount immunostained for the brain-specific homeobox/POU domain protein 3A (Brn3a). (**B**–**H**) Representative high-magnification images from the middle retina (sampling location at about 2.5 mm from the optic disc) from control rats (**B**) or rats that received MCE either untreated (**C**) or treated with vehicles (**D,E**), timolol (**F**), brimonidine (**G**) or melatonin/agomelatine (**H**). Scale bars: 1 mm (**A**) or 150 µm (**B**–**H**). Magnification: 4× (**A**) or 20× (**B**–**H**). (**I**) RGC density based on counting analysis of Brn3a-labeled cells. MCE injection reduced RGC density. Decreased RGC density was unaffected by vehicles, but partially recovered with timolol. After brimonidine or melatonin/agomelatine, RGC density recovered to its control value. Data are shown as box-and-whiskers plots (*n* = 6 retinas for each experimental group). * *p* < 0.05 versus control; ^§^ *p* < 0.05 versus the respective vehicle (one-way ANOVA followed by Tukey’s post hoc test). MelAgo—melatonin/agomelatine.

The activation of the intrinsic apoptotic cascade is a common mechanism leading to RGC loss in human glaucoma and in experimental glaucoma models [40]. We examined whether improved retinal function after brimonidine or melatonin/agomelatine was accompanied by a reduced RGC loss by evaluating the distribution pattern of Brn3a-positive RGCs in retinal flat mounts. Figure 7A represents low magnification image of a control retina. As shown by the representative high magnification images of Figure 7B–H, in comparison to control retinas (Figure 7B), MCE injection resulted in a significant RGC loss (Figure 7C). Treatment with vehicles did not affect the MCE-induced RGC loss (Figure 7D,E). As compared to vehicle-treated retinas, timolol (Figure 7F), brimonidine (Figure 7G), or melatonin/agomelatine (Figure 7H) spared RGCs although with different efficacy as demonstrated by RGC quantification (Figure 7I). MCE reduced the RGC density by about 29%. A comparable loss was observed after MCE in the presence of vehicles, while timolol spared RGC loss by about 11%. Either brimonidine or melatonin/agomelatine spared almost completely RGCs, the density of which did not significantly differ from that in controls. Both the reduction in RGC density and its sparing did not display any retinal regionality (Table 2).

## 3. Discussion

Growing interest in melatonin as a potential therapeutic agent in several diseases stems from its pleiotropic effects. In particular, melatonin shows a protective role in neurological disorders [3] including models of degenerative diseases of the retina. Among retinal diseases, hypertensive glaucoma is characterized by predominant apoptotic death of RGCs further to increased IOP. In this respect, the hypotensive efficacy of melatonin combined with its potential neuroprotective activity would be an important advantage for developing novel approaches to glaucoma therapy.

Although animal models of hypertensive glaucoma are hardly attributable to a given pathological state in humans, studies carried out over the past two decades included a wide variety of possible glaucoma models induced by different mechanisms in rodents. These studies have greatly improved our understanding of the pathophysiology of glaucoma and served as a useful tool mostly in respect to investigating neuroprotective agents. Using a rat model of glaucoma with sustained IOP elevation, our study provides the first evidence that an association of melatonin and agomelatine given as eye drops has a potent hypotensive effect, blunts the inflammatory response in the retina, preventing the massive death of RGC, which is reflected at the functional level by recovered amplitude of both PhNR and PERG likely indicating restored RGC function. In comparison, timolol or brimonidine, two drugs clinically used for the treatment of glaucoma, show a lesser efficacy on IOP reduction. Protective efficacy on RGCs is modest for timolol, but more pronounced for brimonidine, like that of melatonin/agomelatine, though brimonidine shows no efficacy on the inflammatory markers. Protective efficacy on RGCs is reflected at the functional level with negligible effects of timolol on PhNR and PERG, while brimonidine prevents RGC-associated electroretinographic dysfunction similarly to the melatoninergic compounds, thus suggesting a different mode of action.

### 3.1. The MCE Model

The experimental model used here has been previously characterized [17,34,35,36]. In this model, MCE causes a viscosity-dependent ocular hypertension by blocking the trabecular meshwork and preventing the outflow of the aqueous humor, an obstruction that is removed over time; the increased IOP is indeed maintained for about 16 days to progressively decrease in the following period. [36]. As demonstrated here, the retina of the MCE model shows activation of gliotic processes, inflammation and apoptosis. In addition, the reduction in the amplitude of both the PhNR and the PERG suggest a functional deficit in RGCs. In particular, in the PERG, the reduction in the amplitude of the N95 component, which is likely to be generated by action potentials produced by RGCs [42], is in line with previous data in experimental glaucoma and in early glaucoma patients [43,44]. Although the source of the P50 is debated, there is evidence suggesting that also this PERG component is generated by RGCs, with an additional contribution from more distal sites [42]. The presence of a functional deficit in RGCs is also supported by the finding that Brn3a immunoreactivity is markedly reduced after MCE, in line with previous findings in a mouse model of MCE-induced hypertensive glaucoma [34].

### 3.2. Hypotensive and Protective Efficacy of Timolol and Brimonidine

We demonstrated here that timolol induces a consistent reduction of IOP elevation with only limited neuroprotective effects on RGCs as also confirmed by RGC quantitation and by electrophysiological findings showing negligible effects on RGC survival and dysfunction. Timolol belongs to first line therapies used to lower IOP in hypertensive glaucoma. Timolol is a non-selective beta blocker that binds to beta adrenergic receptors, thus blocking their endogenous stimulation and preventing the production of aqueous humor in the ciliary epithelium [45]. Timolol has limited protective efficacy on RGCs; which is mostly dependent on its hypotensive effect [12]. This is in line with the present findings in the MCE model of glaucoma.

An additional IOP-lowering treatment is the alpha 2 adrenergic agonist brimonidine, which acts by activating presynaptic alpha-2 receptors thus leading to decreased catecholamine release and decreased activation of the cAMP pathway, which in turn is coupled to decreased aqueous production by the ciliary body epithelium and reduced resistance to uveoscleral outflow [13]. As shown by the present results, brimonidine reduces the MCE-induced increase in basal IOP by about 30% with an efficacy similar to that of timolol. RGC rescue after timolol is about 11% with respect to the number of RGCs committed to die while MCE-induced RGC death is almost completely prevented by brimonidine. At the functional level, both PhNR and PERG are approximately recovered suggesting the possibility that some part of brimonidine efficacy may be independent on IOP reduction. This is also supported by previous findings demonstrating that brimonidine protects RGCs in models of optic nerve injury, in which IOP has no relevance [46], and in models of normotensive glaucoma [47]. In addition, in models of ocular hypertension, systemic or intraperitoneal brimonidine attenuates RGC death independently on IOP reduction [48]. The direct neuroprotective efficacy of brimonidine may depend on its higher availability to the posterior eye, as demonstrated by previous findings indicating that, after administration of 0.1% eye drops, brimonidine reaches a vitreous concentration of 2 nM [49].

### 3.3. Distribution of Melatonin and Agomelatine

Pharmacokinetics analysis after 2 h from the third instillation of 40 µg of each component in 10 µL per eye shows some important facts. The retention of melatonin and agomelatine is good, amounting to 2.9% and 3.9%, respectively, in line with what could have been expected from bibliographic data [50], considering in our experiments the 2 h of delay between administration and enucleation. The finding that the content of agomelatine in the whole eye (9.94 ng/mg) is 1.33 times higher than that of melatonin (7.44 ng/mg) suggests that agomelatine penetrates the eye more efficiently than melatonin. If we might exclude that the difference is due to differential biodegradation of the molecules, then the difference could be explained by the different retention and penetration of one molecule with respect to the other. The present data also show that the distribution of the two molecules in the posterior segment is strikingly different, hinting also at a different distribution among eye tissues. In the vitreous humor melatonin concentration is 43 nM, eight times lower than agomelatine concentration, which amounts at 332 nM. The difference becomes much higher in the retina, where melatonin concentration is 17 nM, while agomelatine concentration is 378 nM, twenty-two times higher. As a term of comparison, these values do not differ much from what was reported for brimonidine eye drops in monkeys, showing concentration values around 80 nM, while in rats treated by i.p. injection brimonidine values in the vitreous or the retina ranged between 22 and 390 nM, enough to activate the cognate alpha 2-adrenergic receptors [51]. Indeed, since the affinity of MT1 and MT2 melatonin receptors for melatonin is 0.08 and 0.38 nM and for agomelatine is 0.01 and 0.12 nM [52], the concentration present in the posterior segment is more than enough for a consistent stimulation. The reason why melatonin and agomelatine penetrate the eye globe with similar efficiency but distribute differently in eye tissues is unknown. It is reasonable to suppose that agomelatine penetrates in the posterior segment with higher efficiency than melatonin, and this should be dependent on subtle differences in the molecular structure. However, the fact remains that more than 99% of the melatoninergic molecules found in the whole eye globe reside outside the posterior segment, likely within the fibrous layers of cornea, conjunctiva and sclera, from which they might be progressively released into the internal chambers of the eye, and reach their target tissues in the anterior chamber (the trabecular meshwork and/or the ciliary body for IOP regulation) and the retina for neuroprotection. This might explain the long duration (8 h and maybe more) of the IOP effects already reported [17].

### 3.4. Hypotensive and Protective Efficacy of Melatonin/Agomelatine

When applied as eye drops, both melatonin and agomelatine reduce IOP in normotensive and hypertensive eyes with a higher efficacy in the latter [17,18,19,20]. In addition, in glaucoma patients treated by multiple hypotensive topical drugs and under additional treatment with agomelatine for psychiatric problems, oral agomelatine has been shown to further decrease IOP in both eyes whereas multiple drug treatment with anti-glaucoma eye drops had no further effect [22]. As shown here, melatonin/agomelatine reduces the increase in basal IOP by about 60%, an effect that is significantly higher than that of timolol or brimonidine. This higher efficacy of melatoninergic agents may be a consequence of synergistic/additive effects between melatonin and agomelatine as suggested by their longer lasting hypotensive efficacy when administered in combination than when administered alone [17].

The mechanisms through which melatoninergic agents reduce IOP are not fully understood and would involve an increase in the trabecular meshwork outflow through the activation of several pathways eventually coupled to the activation of melatonin receptors which are present in numerous ocular tissues, including the aqueous-humor-producing ciliary processes [16]. In addition, melatonin could act by stimulating antioxidant enzymes or inhibiting prooxidant enzymes, but also by directly scavenging free radicals, thus exerting a potent antioxidant effect that may play an important role in reducing IOP [53].

Both RGC quantitation and functional data shown here concur to demonstrate that melatonin/agomelatine exerts potent protective effects on RGCs that are almost completely spared from death. As demonstrated by the present results, RGC survival is reflected at the functional level by preserved amplitude of both PhNR and PERG likely indicating restored RGC function. A variety of treatments, in addition to conventional IOP-lowering therapies, may be helpful to interfere with progression of the disease. Recently, at the preclinical level, several molecules have been evaluated in the treatment of glaucoma, although most of them failed in the clinical trial. For instance, in a mouse model of spontaneous glaucoma, eye drops based on brain derived neurotrophic factor prevent the reduction in RGC number thus increasing PERG responses [54]. In addition, in open-angle glaucoma patients, topical applications of either the free radical scavenger coenzyme Q10 in conjunction with vitamin E or the choline donor citicoline result in increased amplitude of the PERG P50-P95 wave suggesting a beneficial effect on the inner retinal function [55,56]. RGC survival after melatonin/agomelatine is likely a consequence of drastic IOP reduction although direct effects on RGCs cannot be excluded. In fact, since the late nineties melatonin is known to exert neuroprotective effects in models of RGC degeneration [57], although its efficacy in glaucoma models remained to be fully explored. In the retina, melatonin is believed to exert protective effects on pigment epithelial cells, photoreceptors and ganglion cells [21]. In experimental models of retinopathies, melatonin increases RGC survival in response to an ischemic insult and prevents visual dysfunction in a mouse model of age-related macular degeneration [23,28]. However, in a rat model in which IOP elevation has been obtained through the cauterization of three of four episcleral veins, intraperitoneal injection of melatonin fails to protect RGCs from apoptotic death [30]. This may be due to the fact that systemic administration of melatonin does not provide the eye with an adequate amount of the drug able to exert neuroprotective effects. In addition, difference in the doses and in the glaucoma models may also be responsible for contrasting results obtained in different studies.

### 3.5. Mechanisms Leading to RGC protection

As shown by the present results, neither timolol nor brimonidine affect MCE-induced gliosis-related inflammation with the exception of a modest inhibitory action of brimonidine on GFAP levels. This is in agreement with previous findings demonstrating that brimonidine attenuates the increase in GFAP immunoreactivity in a rat model of chronic ocular hypertension [58]. Although brimonidine has been found to display anti-inflammatory properties in the erythematous skin through the modulation of the vascular barrier function [59], no information is available on its possible anti-inflammatory activity in the diseased eye. Rather, chemotactic properties of various brimonidine formulations in respect to leukocyte migration may determine undesirable inflammatory complications to the eye [60]. Additionally, allergic conjunctivitis and granulomatous uveitis in the elderly can occur after brimonidine administration presumably through activation of T-cells/macrophages [61]. Early papers in models of optic nerve crush demonstrate that brimonidine acts by activating the alpha 2 adrenergic pathway that results in the protection of RGC axons from degeneration [62,63]. In experimental models of glaucoma, alpha 2 adrenergic receptor modulation of glutamate excitoxicity has been proposed as a major mechanism of RGC protection [64,65] dependent or not on brimonidine efficacy on IOP elevation [13]. Brimonidine-induced counteraction of glutamate excitoxicity leads to upregulated expression of the brain-derived neurotrophic factor and fibroblast growth factors at the level of RGCs, thus intervening on the apoptotic cascade leading to RGC loss [65,66,67]. Brimonidine-induced production of amyloid beta has been proposed as an additional mechanism through which brimonidine spares RGCs from apoptotic death independently on IOP reduction [68].

Müller cells are the major type of non-neuronal cells in the vertebrate retina. Müller cells normally do not express the glial specific protein GFAP, which is instead accumulated in response to neuronal injury and degeneration, both leading to gliosis-related inflammatory processes. In particular, activated microglia in response to inflammation is able to influence Müller cells response that may serve to augment initial inflammatory responses. The exact mechanisms by which retinal ganglion cell axons are insulted and finally degenerate in glaucoma are not known. However, glaucoma-related stimuli, including ocular hypertension, may induce Müller cells to become reactive and hypertrophic and able to produce mediators, such as cytokines and interleukins, which may affect neuronal survival [69]. The present finding that melatonin/agomelatine drastically reduces GFAP levels in the retina together with the fact that GFAP is an important marker of Müller cell activation prompt us to assume that melatoninergic agents may have an important impact on Müller cell activation. In this respect, treatments counteracting neuroinflammation are known to reduce gliosis and Müller cell activation, an effect that is highlighted by a reduced GFAP staining. As an example, in a rat model of hypertensive glaucoma, neuroprotective treatments have been shown to reduce both Müller cell activation and GFAP levels [70]. Accordingly, it is likely that the melatonin/agomelatine-induced reduction of GFAP levels may be paralleled by a reduction in Müller cell activation. As shown by the present results, melatonin/agomelatine protects RGCs by inhibiting gliosis-related inflammation thus preventing the apoptotic cascade leading to RGC loss. Inhibiting gliotic processes by Müller cells, which are known to express melatonin receptors [71], may serve to increase RGC survival. In the glaucomatous retina, in fact, activated glial cells participate to the production of the inflammatory milieu that plays a major role in RGC degeneration [69]. As shown here, the imbalance in the production of pro- and anti-inflammatory cytokines may be reverted by melatoninergic compounds suggesting that melatonin and its derivatives may be of help in maintaining the homeostasis of the retinal environment. Melatonin has been shown to rescue the retina from neuroinflammation through inhibiting cytokine production in rodent models of retinal degeneration [53,72,73]. Of note, both endogenous melatonin and IL-4 prevent the production of inflammatory mediators suggesting that they may participate to protect the retina in inflammatory diseases [74]. Anti-apoptotic processes leading to RGC protection occur downstream to the ameliorative effect of melatoninergic compounds on gliosis-related inflammation, although anti-apoptotic efficacy through a direct action on RGCs cannot be excluded. In retinal pigment epithelial cells, for instance, melatonin blocks experimentally induced apoptosis by acting directly at its receptors [75,76]. Downstream to its activation, MT1, which is coupled to a Gi protein, inhibits forskolin-stimulated cAMP, protein kinase A signaling, and CREB phosphorylation. MT1 also induces the phosphorylation of mitogen-activated protein kinase 1/2 and extracellular signal–regulated kinase 1/2, and increase potassium conductance through Kir inwardly rectifying channels. MT2 is also coupled to a Gi protein and inhibits both forskolin-stimulated cAMP production and cGMP formation. In addition, it activates protein kinase C in the suprachiasmatic nucleus, and decreases calcium-dependent dopamine release in the retina [52]. Downstream to these pathways, receptor-mediated effects of melatonin include the inhibition of inflammatory cytokine production, the release of neurotrophic factors targeting RGCs and the reduction of glutamate excitotoxicity thus ultimately resulting in the prevention of apoptotic death of RGCs. The intracellular signaling pathways responsible for these effects of melatonin are still under investigation.

## 4. Materials and Methods

### 4.1. Animals

The present study was performed in agreement with the ARVO Statement for the Use of Animals in Ophthalmic and Vision Research and the Guide for the Care and Use of Laboratory Animals of the National Institutes of Health. This study also adheres to the European Communities Council Directive (2010/63/UE) and the Italian guidelines for animal care (DL 26/14) and was approved by the Commission for Animal Wellbeing of the University of Pisa (permit number 133/2019-PR, 14 February 2019). The number of rats used in the present study, as well as their suffering, was limited according to the 3Rs principles for ethical use of animals in scientific research. Rats (eight weeks old, males and females) of the Sprague–Dawley strain were from Charles River Laboratories Italy (Calco, Italy), and were maintained under standard laboratory conditions of 12-h cycle of light and dark (lights on at 8 a.m.), with free access to food and water. All rats were acclimatized for 1 week to handling and tonometry before starting the study. Sixty-nine rats (36 males and 33 females) were used. Of them, six rats were used for the pharmacokinetics study. The additional 63 rats were divided in seven experimental groups (9 rats/experimental group). One group was used as normal control, while the other groups were injected with MCE. Of them, one group was left untreated, while the other groups were treated with eye drops based on timolol, brimonidine, and melatonin/agomelatine or their vehicles.

### 4.2. Preparation of Eye Drops Containing Melatonin/Agomelatine

Eye drops containing melatoninergic compounds (0.4% melatonin and 0.4% agomelatine) were prepared in nanomicellar formulation (11.5% Soluplus in Tris buffer pH 7.4 adjusted to 300 mOsm with NaCl) with the addition of 0.1% lipoic acid, as previously described [17]. The nanomicellar formulation was used to increase the corneal residence time and to favor corneal permeation [77], while lipoic acid was used as a stabilizing excipient to protect melatoninergic agents from oxidation thus increasing their half-life [78].

### 4.3. Pharmacokinetics Study

Eye drops containing melatonin/agomelatine (10 µL) were given into each eye of five rats while an additional untreated rat was used to generate a standard calibration curve. Eye drops administration (three times at the distance of 2 h between each instillation) started at 10 a.m., 2 h after lights on in the vivarium, a time known to be sufficient to reduce the production of endogenous melatonin to its minimum in the rat [79]. Two hours after the last treatment, animals were euthanized with an overdose of pentobarbital and the eye globes removed. One eye of each rat was taken whole, while the fellow eyes were dissected, and retinas and vitreous humors were separately taken. Whole eyes were cut in small pieces and separately homogenized with an Ultra-turrax homogenizer for 3 min on ice at 4 °C in 0.25 mL of methanol, then subjected to 1 min of ultrasonication in a cold-water bath. The five retinas were pooled together and extracted as before in 0.1 mL of methanol. Each vitreous body was diluted with 0.05 mL of methanol and sonicated. All samples were finally centrifuged 10 min at 10,000× *g* rpm and the supernatants were saved frozen for further analysis. Melatonin and agomelatine were quantitated in tissue extracts by HPLC/MS/MS using the triple quadrupole instrument Agilent 6410-A equipped with a Phenomenex Kinetex C18 column at 25 °C under isocratic conditions using 30% of Buffer A (5 mM ammonium formate, 0.1% formic acid) and 70% of methanol at a flow rate of 0.1 mL/min. The system is equipped with a positive ionizing mode ESI interface, such that the mass transition for melatonin is 233.2 → 216.2 m/z and for agomelatine is 244.1 → 185.3 m/z [80]. The operational MS parameters of the instrument were: gas temperature, 350 °C; gas flow, 5 L/min; nebulizer, 20 psi; capillary, 3500 V; collision energy, 15 V; dwell-time, 200 msec; fragmentor, 135 V.

To generate a standard calibration curve, whole eye extract was added with exogenous purified molecules at concentrations between 1 and 200 ng/mL for melatonin (R^2^ > 0.993), and 1 and 500 ng/mL for agomelatine (R^2^ > 0.999). The linearity and correlation coefficient of such standard curves hint that no endogenous melatonin or agomelatine were detectable in the samples and did not interfere with the quantitative analysis. In particular, we sacrificed the rats at 4 p.m., when the production of endogenous melatonin is at its minimum, just to avoid any interference in the pharmacokinetics study.

### 4.4. Experimental Model of Intraocular Hypertension

The model was originally developed in rabbits [36] and then applied to rodents [17,34,35]. It is based on the injection of 2% MCE in sterile saline in the anterior chamber of the eye. The MCE used in the present study (M0512; Sigma Aldrich, St. Louis, MO, USA) is pure and has a molecular weight of about 88,000 and, when dissolved in aqueous solution at 2% gives a solution with a viscosity ranging from 3500 to 5600 cps. Rats, anesthetized with an intraperitoneal injection of pentobarbital (30 mg/kg), were injected into the anterior chamber with 15 µL of MCE in both eyes using a 18G needle in agreement with published protocols [17,35]. As previously reported, following MCE injection, a transient rise in IOP due to the increased volume of the aqueous humor after the injection could be observed [17]. After returning to its basal level within 1 h, IOP reached a second peak within 4 h after MCE administration as a consequence of the trabecular tissue clotting; then, 8 h after MCE injection IOP was stabilized to about 30 mmHg [17,35]. The eyes of each rat were evaluated for unintended adverse effects of intraocular injection -e.g., cataract, corneal opacity or edema that could impact electrophysiological parameters and/or tonometer accuracy.

### 4.5. Eye Drop Administration

Twenty-four hours after MCE injection, IOP elevation was assessed and the rats were subjected to topical applications of eye drops containing saline (vehicle 1), nanomicellar formulation (vehicle 2), timolol (Timoptol 0.5%^®^; Santen Italy, Milano, Italy), brimonidine (Brimonidina Sandoz 2 mg/mL^®^; Sandoz SpA, Origgio, Italy) or melatonin/agomelatine. Eye drops (10 µL) were administered to both eyes two times a day over two weeks after MCE injection. Eye drops were instilled at 10 a.m. and at 6 p.m. The regimen of the treatments was in line with previous findings demonstrating hypotensive efficacy of the mixture [17]. After melatonin/agomelatine no evident signs of ocular allergy, hyperemia, or discomfort were observed. This is in agreement with previous findings in this same model [17] in which no evidence of corneal or conjunctival toxicity, such as signs of chemical trauma, iatrogenic corneal toxicity, inflammation, or conjunctivitis, were observed after eye drop applications. This is also in line with previous findings demonstrating that eye drops based on melatoninergic agents did not induce toxicity in a battery of standard ocular surface irritation studies [81].

### 4.6. IOP Measurement and Quantitation

To measure IOP, rats were restrained in a soft plastic cone and secured. IOP of both eyes was measured by an operator masked to treatment status using a TonoLab tonometer (Icare, Vantaa, Finland) following manufacturer’s recommendations. Measurements were performed over three days before MCE administration, the same day of MCE administration and at days 1, 3, 6, 9, 12, and 15 after MCE administration. Measurements were taken at 10 a.m., just before eye drop instillation. Mean IOP (average of 10 consecutive measurements) over the experimental period was calculated for all individual eyes from each group.

### 4.7. Evaluation of Retinal Function

Full field photopic ERG was recorded in control rats and in rats that received MCE, untreated or treated with eye drops based on vehicles, timolol, brimonidine, or melatonin/agomelatine. RGC function was evaluated by measuring the PhNR as previously described [34]. Electroretinographic recordings were made using a Ganzfeld stimulator (Biomedica Mangoni, Pisa, Italy). Rats were dark adapted overnight and anesthetized with an intraperitoneal injection of pentobarbital (30 mg/kg). The electroretinographic responses were recorded using Ag/AgCl corneal electrodes. A reference electrode was placed on the forehead while a ground electrode was placed on the tail. After anesthesia, rat pupils were dilated with 0.5% atropine and the cornea was intermittently irrigated with saline solution to prevent clouding of the ocular media. Photopic cone-mediated responses were recorded at the light intensity of 3 cd-s/m^2^ in rats that were light-adapted for 10 min on a background light intensity of 30 cd/m^2^ to suppress the rod response. Photopic ERG recording was performed from both eyes simultaneously. For each rat, 10 waveforms were recorded with an interstimulus interval of 3 s and averaged. The light intensity of 3 cd-s/m^2^ elicits a negative a-wave, mainly generated by cones, and a positive b-wave, generated by post-photoreceptor cells. In the photopic ERG, the PhNR, which is mainly driven by RGCs, was identified as the first negative deflection after the b-wave, calculating its amplitude relative to baseline (0 μV).

PERG responses, mainly generated by RGC activity, can be useful for assessing RGC function in conditions such as glaucoma and ischemic optic neuropathy [82]. PERG responses were evoked using an alternating pattern of black and white horizontal bars delivered on a stimulus display unit from a commercially available PERG system (SB700 Advanced, Nikon-Europe, Amsterdam, The Netherlands). Stimuli consisted in 0.05 cycles/deg black and white bars reversing at 1 Hz presented at 98% contrast. The pattern stimuli were administered through a light emitting diode display with a mean luminance of 50 cd/m^2^ aligned at about 20 cm from the corneal surface. A total of 200 signals were averaged. Each animal was placed at the same fixed position in front of the monitor to prevent recording variability due to animal placement. PERG recordings were sequentially conducted in both eyes. The PERG response was evaluated by measuring the amplitude of the N35-P50 wave (from the trough of the negative peak N35, a small deflection near 35 ms after the stimulus, to the peak of the positive peak P50, a deflection near 50 ms after the stimulus) and the P50-N95 wave (from the peak of the positive peak P50 to the trough of the negative peak N95, a deflection near 95 ms after the stimulus). The implicit time was determined by measuring the time from the onset of the stimulus to the P50 and N95 peaks.

### 4.8. Western Blot Analysis

After animal euthanization with an overdose of pentobarbital, retinas were isolated and stored at −80 °C. Six samples per group, each containing two retinas from independent rats, were used. RIPA extraction buffer containing phosphatase and proteinase inhibitor cocktails (Roche Applied Science, Indianapolis, IN, USA) was used to homogenize each sample. Protein concentration was measured using the Micro BCA Protein Assay (Thermo Fisher Scientific, Waltham, MA, USA). Thirty micrograms of proteins from each sample were run on 4–20% SDS-PAGE gels before transferring proteins on polyvinylidene difluoride membranes. Blots were blocked for 1 h with 5% skim milk and incubated overnight at 4 °C with the primary antibodies listed in Supplementary Table S1 that are routinely used to detect the inflammatory and the apoptotic markers as determined in the present study. β-actin was used as loading control. Blots were then incubated for 1 h with the appropriate HRP-conjugated secondary antibodies (1:5000 dilution) and developed with the Clarity Western enhanced chemiluminescence substrate (Bio-Rad Laboratories, Inc., Hercules, CA, USA). Images were then acquired (ChemiDoc XRS+; Bio-Rad Laboratories, Inc., Hercules, CA, USA). The optical density (OD) of the bands was evaluated (Image Lab 3.0 software; Bio-Rad Laboratories, Inc., Hercules, CA, USA) and data were normalized to the corresponding OD of β-actin or NF-κB p65 as appropriate. All evaluations were performed in duplicate.

### 4.9. Retinal Ganglion Cell Immunohistochemistry and Quantitation

After animal euthanization with an overdose of pentobarbital, six retinas from independent rats for each experimental group were explanted. Retinas were then fixed for 90 min at 4 °C in 4% paraformaldehyde in 0.1 M phosphate buffer (PB) and stored at 4 °C in 25% sucrose in 0.1 M PB. Retinal ganglion cells were labeled incubating the retinas for 24 h at 4 °C with a mouse monoclonal antibody against Brn3a (1:100 in PB containing 5% BSA and 2% TritonX-100; sc-8429; Santa Cruz Biotechnology, Santa Cruz, CA, USA). Retina whole mounts were then washed with PB and incubated overnight at 4 °C in an AlexaFluor555-conjugated secondary antibody (1:100; A-32727; Molecular Probes, Eugene, OR, USA). Finally, retinas were washed with PB, mounted on glass slides with the photoreceptor side facing down and viewed with a fluorescence microscope (Ni-E; Nikon-Europe, Amsterdam, The Netherlands). Images of the entire retina were acquired by a DS-Fi1c camera (Nikon-Europe) and Brn3a-positive cells were counted using the NIS-Elements software (Nikon-Europe). The mean RGC density was determined by averaging the count of Brn3a-positive cells in three areas (500 × 500 μm) per retinal quadrant (superior, inferior, nasal and temporal) at three different distances from the optic disc (1–2 mm, central retina; 2–3 mm, middle retina; 3–4 mm, peripheral retina). In total, 12 images were taken from each retina. The density of Brn3a-positive cells (number of cells per mm^2^) was compared between the different experimental groups. Quantification was performed in a masked manner.

### 4.10. Data Analysis

Data were analyzed by the Shapiro–Wilk test to verify their normal distribution. Statistical significance was evaluated with Prism 8.0.2 (GraphPad Software, Inc., San Diego, CA, USA) using one-way ANOVA followed by Tukey post-hoc test or two-way ANOVA followed by Bonferroni’s multiple comparison post-test. Vehicle 1 (saline) was used to compare data obtained after timolol or brimonidine, while vehicle 2 (nanomicellar formulation) was used to compare data obtained after melatonin/agomelatine. In Figure 1 and in Table 1 and Table 2, data are expressed as means ± SEM, while in Figure 2, Figure 3, Figure 4, Figure 5, Figure 6 and Figure 7, data are expressed as box and whiskers plots, with boxes representing the 25th–75th percentiles with median, and whiskers indicating minimum and maximum values. Differences with *p* < 0.05 were considered significant.

## 5. Conclusions

Interventions intended to lower IOP, the most common risk factor for glaucoma progression, are not always effective in preventing visual field loss. Therefore, therapeutical approaches that combine hypotensive efficacy with neuroprotective effects on RGCs should play a role in the future of glaucoma treatment. In the present study, we demonstrated that in a glaucoma model, a nanomicellar ophthalmic formulation of melatonin/agomelatine besides exerting a potent hypotensive effect, is also effective in counteracting RGC loss and recovering RGC function through an inhibition of gliosis-related inflammation. On the basis of the present results, it is entirely possible, if not probable, that the protective efficacy of melatonin/agomelatine on RGCs is secondary to IOP reduction although the possibility of a direct neuroprotection cannot be excluded. Based on the similarity of the effects of melatonin/agomelatine with those of brimonidine, it is plausible to argue that the combination of melatonin and agomelatine may have a direct neuroprotective activity similarly to brimonidine. On the other hand, the higher hypotensive efficacy of melatonin/agomelatine in respect to that of timolol or brimonidine is a confounding factor that does not allow us to differentiate with confidence direct and indirect neuroprotective effects exerted by the melatoninergic compounds. In this respect, much work remains to be done in order to dissect a direct protective efficacy of melatoninergic compounds from indirect effects consequent to IOP lowering.

**Figure 8:**
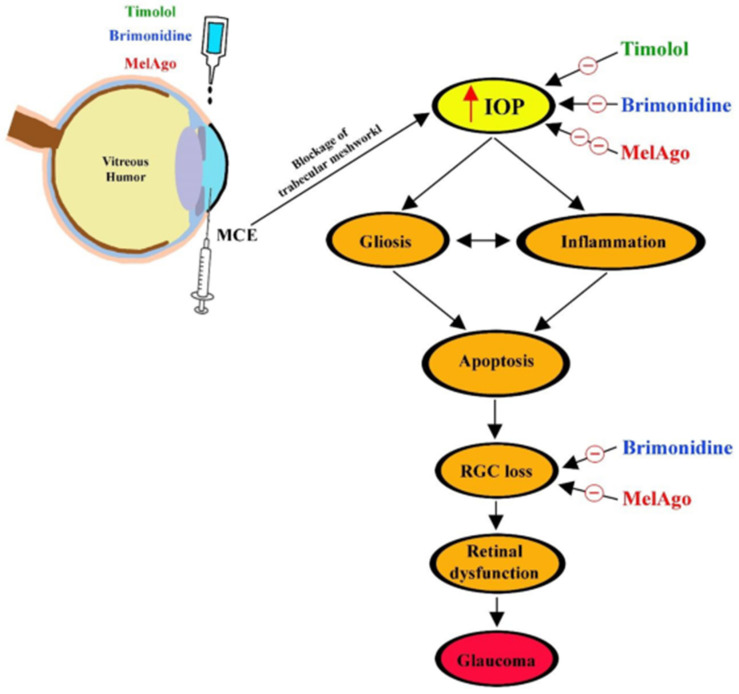
Schematic diagram depicting the effects of timolol, brimonidine, and melatonin/agomelatine in the MCE model of hypertensive glaucoma. MCE blocks the trabecular meshwork, thus preventing the outflow of the aqueous humor, which causes IOP elevation (red arrow). In turn, IOP increase activates gliotic and inflammatory processes that induce apoptotic death of retinal cells, including RGCs. RGC loss leads to retinal dysfunction, which is a feature of hypertensive glaucoma. Timolol acts mainly by reducing IOP, while brimonidine and melatonin/agomelatine (MelAgo) also spare RGCs from apoptotic death, thus leading to recovered retinal dysfunction.

The schematic diagram of Figure 8 summarizes the cascade of events leading to RGC loss in the MCE model of hypertensive glaucoma. Each step of the cascade is restored by melatonin/agomelatine. Timolol mostly acts on IOP increase, while brimonidine also restores RGC density and function, but its hypotensive efficacy is lower than that of melatonin/agomelatine.

In conclusion, results of the present study in a glaucoma model suggest that exogenous melatonin/agomelatine applied as eye drops may be beneficial for ocular health although additional investigations and clinical data are needed to establish its therapeutic potential.

## Figures and Tables

**Table 1:** Concentration of melatoninergic compounds in the whole eye, the retina, and the vitreous.

			Melatonin			Agomelatine		
Animal	Sample Type	Weight (mg)	Concentration (ng/mg)	Mean	SEM	Concentration (ng/mg)	Mean	SEM
1	Whole eye	151	7.83	7.44	0.85	10.89	9.94	0.68
2	Whole eye	165	10.21			10.64		
3	Whole eye	176	7.87			11.35		
4	Whole eye	160	5.64			9.16		
5	Whole eye	150	5.67			7.67		
1 to 5	Pool of retinas	115	0.004	-	-	0.096		
1	Vitreous	11	0.007	0.010	0.001	0.071	0.081	0.005
2	Vitreous	12	0.008			0.073		
3	Vitreous	10	0.013			0.099		
4	Vitreous	13	0.010			0.077		
5	Vitreous	11	0.014			0.084		

**Table 2:** Effect of timolol, brimonidine, or melatonin/agomelatine on RGC density in central, middle, and peripheral retina.

	RGC density (% of control)		
	Central	Middle	Peripheral
**Control**	100 ± 4	100 ± 5	100 ± 7
**MCE**	70 ± 6 *	71 ± 4 *	71 ± 6 *
**+ Vehicle 1**	72 ± 5 *	72 ± 3 *	70 ± 4 *
**+ Vehicle 2**	73 ± 7 *	72 ± 4 *	71 ± 6 *
**+ Timolol**	78 ± 4 *^,§^	83 ± 3 *^,§^	81 ± 4 *^,§^
**+ Brimonidine**	88 ± 3 ^§^	91 ± 6 ^§^	91 ± 3 ^§^
**+ MelAgo**	92 ± 5 ^§^	94 ± 5 ^§^	94 ± 3 ^§^

Data are shown as mean ± SEM (*n* = 6 for each experimental group). * *p* < 0.05 versus control; ^§^ *p* < 0.05 versus the respective vehicle (one-way ANOVA followed by Tukey post-hoc test). The RGC density in the central, middle and peripheral retina was 2365 ± 205/mm^2^, 2175 ± 315/mm^2^, and 1657 ± 325/mm^2^, respectively, as previously reported [41]. MelAgo, melatonin/agomelatine.

## Supplementary Materials

The following are available online at https://www.mdpi.com/1422-0067/21/23/9267/s1.

## Abbreviations

Brn3a	Brain-specific homeobox/POU domain protein 3A
ERG	Electroretinogram
GFAP	Glial fibrillary acidic protein
Iba1	Ionized calcium binding adaptor molecule 1
IL	Interleukin
IOP	Intraocular pressure
MCE	Methylcellulose
OD	Optical density
PB	Phosphate buffer
PERG	Pattern electroretinogram
PhNR	Photopic negative response
RGC	Retinal ganglion cell
TNF	Tumor necrosis factor
