# Correction: Dal Monte et al. A Topical Formulation of Melatoninergic Compounds Exerts Strong Hypotensive and Neuroprotective Effects in a Rat Model of Hypertensive Glaucoma. *Int. J. Mol. Sci.* 2020, *21*, 9267

**DOI:** 10.3390/ijms27052351

**Published:** 2026-03-03

**Authors:** Massimo Dal Monte, Maurizio Cammalleri, Rosario Amato, Salvatore Pezzino, Roberta Corsaro, Paola Bagnoli, Dario Rusciano

**Affiliations:** 1 Department of Biology, University of Pisa, Via San Zeno, 31, 56127 Pisa, Italy; maurizio.cammalleri@unipi.it (M.C.); rosario.amato@biologia.unipi.it (R.A.); paola.bagnoli@unipi.it (P.B.); 2 Sooft Research Center c/o, University of Catania, Via Santa Sofia, 89, 95123 Catania, Italy; salvatore.pezzino@sooft.it (S.P.); roberta.corsaro@sooft.it (R.C.); dario.rusciano@sooft.it (D.R.)

In the original publication [1], there was a mistake in Figure 7 as published. During the preparation of the manuscript, a draft copy of the figure was inadvertently uploaded instead of the finalized figure intended for publication. The authors apologize for this inconvenience. The corrected Figure 7 appears below. The authors state that the scientific conclusions are unaffected. This correction was approved by the Academic Editor. The original publication has also been updated.

## Figures and Tables

**Figure 7:**
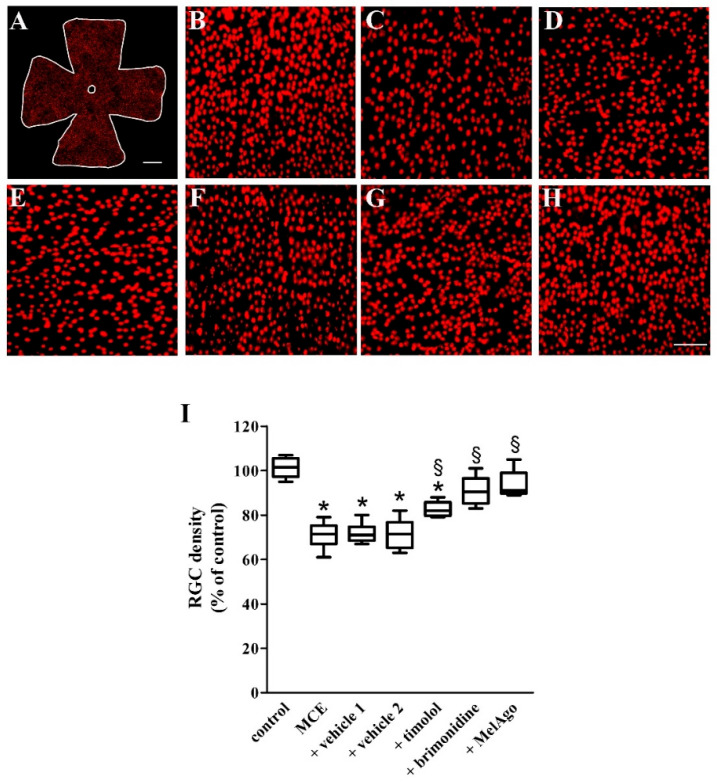
Retinal ganglion cell (RGC) density. (**A**) Representative image of a control flat mount immunostained for the brain-specific homeobox/POU domain protein 3A (Brn3a). (**B**–**H**) Representative high-magnification images from the middle retina (sampling location at about 2.5 mm from the optic disc) from control rats (**B**) or rats that received MCE either untreated (**C**) or treated with vehicles (**D**,**E**), timolol (**F**), brimonidine (**G**) or melatonin/agomelatine (**H**). Scale bars: 1 mm (**A**) or 150 µm (**B**–**H**). Magnification: 4× (**A**) or 20× (**B**–**H**). (**I**) RGC density based on counting analysis of Brn3a-labeled cells. MCE injection reduced RGC density. Decreased RGC density was unaffected by vehicles, but partially recovered with timolol. After brimonidine or melatonin/agomelatine, RGC density recovered to its control value. Data are shown as box-and-whiskers plots (*n* = 6 retinas for each experimental group). * *p* < 0.05 versus control; ^§^ *p* < 0.05 versus the respective vehicle (one-way ANOVA followed by Tukey’s post hoc test). MelAgo—melatonin/agomelatine.
