# Supplementary material for: A Topical Formulation of Melatoninergic Compounds Exerts Strong Hypotensive and Neuroprotective Effects in a Rat Model of Hypertensive Glaucoma
Source: Int J Mol Sci. 2020 Dec 4;21(23):9267. doi: 10.3390/ijms21239267 (PMC7730513; doi:10.3390/ijms21239267)
Supplement: Supplementary file 1 [file ijms-21-09267-s001.pdf]

Table S1. List of antibodies used in Western blot.

Antibody	Dilution	Source	Cat. No.
Rabbit monoclonal anti-Iba1	1:1000	Abcam	ab178846
Mouse monoclonal anti-GFAP	1:1000	Sigma-Aldrich	G3893
Rabbit polyclonal anti-TNF-α	1:1000	Abcam	ab6671
Rabbit polyclonal anti-IL-1β	1:1000	Abcam	ab9722
Rabbit polyclonal anti-IL-6	1:200	Abcam	ab6672
Rabbit polyclonal anti-IL-4	1:500	Abcam	ab9622
Goat polyclonal anti-IL-10	1:100	Santa Cruz Biotechnology	sc-1783
Rabbit polyclonal anti-Bax	1:100	Santa Cruz Biotechnology	sc-493
Rabbit polyclonal anti-Bcl-2	1:100	Santa Cruz Biotechnology	sc-492
Rabbit monoclonal anti-cleaved caspase 3	1:1000	Cell Signaling Technology	9664
Mouse monoclonal anti-β-actin	1:2500	Sigma-Aldrich	A2228
